# Altered neural processing of evaluative feedback from presumed conscious AI

**DOI:** 10.1016/j.isci.2026.117175

**Published:** 2026-08-11

**Authors:** Antje Peters, Merit Ohlsen, Jens Bölte, Su Arkun, Laura Brockhoff, Thomas Straube, Sebastian Schindler

**Affiliations:** 1 Institute for Medical Psychology and Systems Neuroscience, University of Münster, Münster, Germany; 2 Otto Creutzfeldt Center for Cognitive and Behavioral Neuroscience, University of Muenster, Münster, North Rhine-Westphalia, Germany; 3 Goethe University Frankfurt, Epilepsy Center Frankfurt Rhine-Main, Department of Neurology, University Hospital Frankfurt, Frankfurt, Germany; 4 Institute of Psychology, University of Muenster, Münster, North Rhine-Westphalia, Germany

**Keywords:** Social evaluative feedback, machine consciousness, EEG/ERP, valence

## Abstract

Interacting with artificial intelligence (AI) is increasingly prominent in everyday life. Modern AI can produce intelligent, witty, and even sarcastic responses, leading people to assume AI is conscious. This raises the question whether AI is perceived as conscious, and how people respond to such machines behaviorally and neurally. This preregistered electroencephalogram (EEG) study investigated the processing of social evaluative feedback from a human and two putative AI algorithms differing in attributed consciousness. Participants (*N* = 40) received feedback, supposedly sent by either another human, a conventional AI based on a large language model (ChatGPT), or an innovative AI capable of conscious processing (AICA-2). Human received the highest conscious ratings, while AICA-2 was rated significantly higher than ChatGPT. Event-related potentials showed enlarged early posterior negativity (EPN) and late positive potential responses to human vs. AI feedback. Exploratory analyses revealed differences between AICA-2 and ChatGPT, whereas the EPN effect correlated with attributed consciousness.

## Highlights

• Experimentally manipulated AI characteristics influence neural feedback processing
• People perceive AI as potentially conscious
• EPN effects correlate with attributed consciousness

## Graphical abstract

**Figure:**
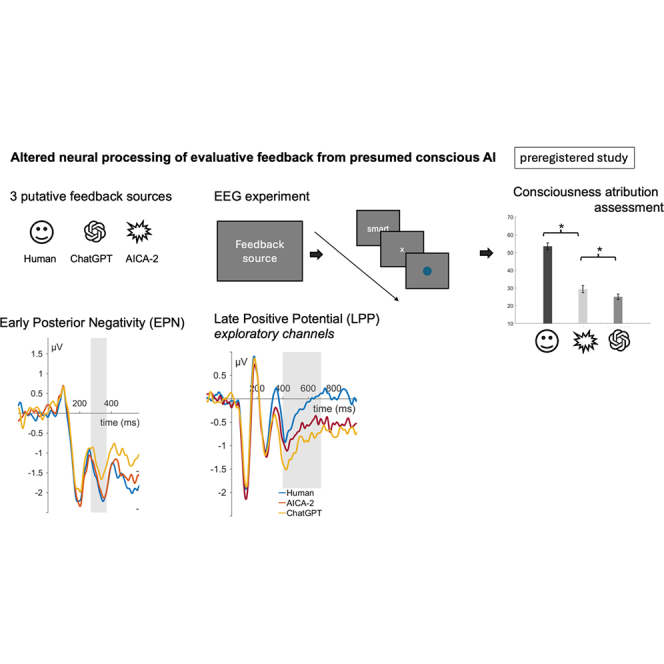

## Teaser

Neuroscience; Artificial intelligence; Human-computer interaction

## Introduction

Chatbots powered by large language models (LLMs) are increasingly prominent in our everyday lives. By 2025, approximately 700 million people globally were using the market-leading ChatGPT every week.^1^ Among students worldwide, more than 70% regularly use AI tools such as ChatGPT.^2^ These models’ ability to reason logically, classify, and answer questions resembles human reactions. The question of whether being polite to an artificial intelligence (AI) agent during a chat session, given its human-like impression, is of great economic and social interest.^3^ It has been shown that users presume a degree of consciousness in LLMs and other interactive technologies.^4^ In fact, they are more likely to be perceived as conscious when they exhibit human-like characteristics, such as physical appearance and cognitive traits such as intelligence, natural language processing, emotional expressions, or self-awareness.^5^

An individual’s consciousness can be examined along two key dimensions: agency and experience.^6^ While the term agency is defined as the capability to plan, control, make decisions, and adapt to learning,^7^ experience is understood as the presence of mental states, including feelings and emotions, that direct and coordinate behaviors.^8^ Users may attribute consciousness to technologies that display either of these dimensions, treating them as if they were human. Therefore, we are confronted with a new type of social partner that performs tasks for us and also serves as an advisory function.^9^ For example, negative computer feedback is more readily accepted than human feedback,^10^ and there is less desire to punish negative decisions when they result from an algorithm^11^ – it might be speculated that lower attribution of experience or intentionality may explain these effects. Trust in computer systems seems to depend on many aspects, including attributed competence and warmth.^10^ Still, it is not yet clear what effects the daily confrontation with assumed conscious machines has if these machines evaluate the user. Given that social interaction is a primary human motivation and fundamental to self-development,^11^ the effects of social feedback from presumed conscious machines should be investigated.

The impact of attributed human compared with artificial computer feedback on brain responses has gained increased attention in neuroscientific research. Still, an unanswered question remains: how does social evaluative feedback from assumed human senders, compared with that from supposed conscious and unconscious machines, affect event-related potentials (ERPs) in the EEG? To address this, previous studies have mapped the temporal course of social evaluative feedback processing, identifying specific ERPs relevant to initial perception as well as intermediate and late stages of information integration. Specifically, the early posterior negativity (EPN), which begins around 200 ms post-stimulus, indexes early top-down or stimulus-driven attention processes for verbal stimuli.^12^ Following this, during the late positive potential (LPP), which occurs approximately 400 ms post-stimulus, more elaborate stimulus processing takes place, including stimulus evaluation, explicit emotional appraisal, self-referential processing, and information integration.^13^^,^^14^^,^^15^ Sender characteristics also appear to influence ERP modulation in response to social evaluative feedback.^16^ Here, robustly, feedback from another human, as compared with randomly acting computers or even to computer algorithms providing informative evaluative feedback, has been shown to alter processing,^16^^,^^17^^,^^18^^,^^19^ for review, see Peters et al.^20^ Regarding feedback valence, both negative and positive feedbacks have been shown to increase EPN and LPP responses, with typically a somewhat larger increase for positive social-evaluative feedback.^17^^,^^18^^,^^21^ Even in cases of identical human feedback, the attribution of sender characteristics, such as expertise, affects feedback processing.^16^ However, comparisons between socially intelligent algorithms and humans have highlighted that the sender’s “humanness” remains a key factor in driving feedback processing.^18^ This raises the question of whether the experimentally induced characterization of the feedback source’s attributed consciousness plays a crucial role in the cognitive processing of socially evaluative feedback and whether this is reflected in enhanced ERPs to feedback.

**Figure 1:**
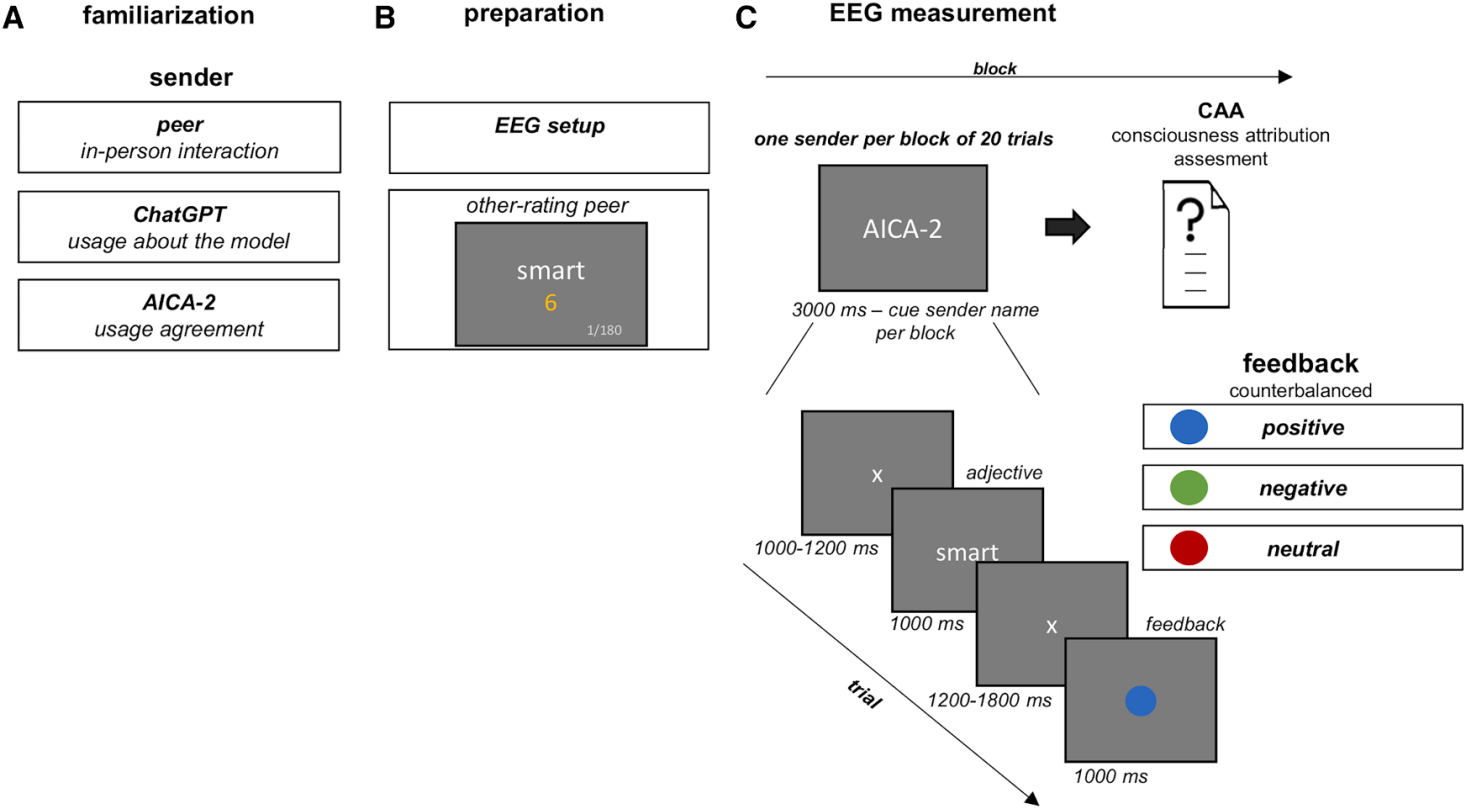
Experimental procedure (A) Participants were familiarized with the three feedback sources, including a real interaction with another human. (B) Participants were prepared for the EEG measurement. In the meantime, participants rated the other participant based on the interaction and were familiarized with the feedback symbols. (C) EEG data were recorded. The experiment began with the feedback source for the next 20 trials, followed by the presentation of the adjective first, then a feedback decision. After each block, the consciousness attribution assessment was performed.

This preregistered study (https://osf.io/hb4cx) investigated whether the experimentally induced characterization of the AI sources influences the processing of social evaluative feedback and whether this is reflected in the neural processing measured by EEG. To this end, we manipulated the ascribed consciousness for the different feedback sources, instructing one to be human, another to be a non-conscious AI agent, and a third to be a conscious AI agent, while in reality, all feedback was random. For an overview of the used stimuli, cf. Table 1. After each block, the ascribed consciousness was assessed to determine whether the instruction was effective (see Figure 1). We registered that when our manipulation is successful, the human sender will receive the highest rating for consciousness, followed by AICA-2, and then ChatGPT. Furthermore, for rated feedback valence, we expected a main effect of valence, with more positive ratings for positive feedback and more negative ratings for negative feedback, and additionally an interaction showing that these effects are pronounced within the human sender, followed by AICA-2 and then ChatGPT. For arousal ratings, we expected separate main effects: higher arousal for human feedback, followed by AICA-2, and then ChatGPT feedback, and for positive and negative feedback compared with neutral feedback. Concerning ERPs, we hypothesized that the strongest EPN and LPP responses would be observed for an attributed human sender, but, importantly, we also expected increased ERPs for AICA-2 compared with ChatGPT, reflecting the experimentally induced characterization of the AI sources. As with arousal, we predicted larger EPN and LPP responses to positive and negative feedback than to neutral feedback.

**Table 1:** Comparisons of the three word lists by one-way ANOVA

Variable	List 1 (*N* = 60)	List 2 (*N* = 60)	List 3 (*N* = 60)	*F* value	*p* value
Valence	5.27 (2.26)	5.29 (2.32)	5.37 (2.33)	0.04	0.966
Arousal	4.01 (0.67)	4.10 (0.71)	3.97 (0.75)	0.53	0.587
Self-relevance	5.48 (1.16)	5.57 (1.19)	5.54 (1.19)	0.10	0.904
Concreteness	5.98 (0.94)	6.16 (0.98)	6.18 (0.93)	0.84	0.436
Word length	9.82 (2.02)	9.88 (1.91)	9.95 (1.62)	0.08	0.926
Word frequency	262.95 (361.21)	255.15 (319.65)	257.82 (356.52)	0.01	0.992
Regularity	118.70 (256.08)	92.52 (152.53)	126.90 (239.72)	0.40	0.673

Values are represented as mean (SD). Valence: 1 = highly negative, 5 = neutral, 9 = highly positive; Arousal 1 = very low, 9 = very high; Concreteness 1 = very concrete, 9 = very abstract. Word frequency is depicted per million.

## Results

### Ratings

**Figure 2:**
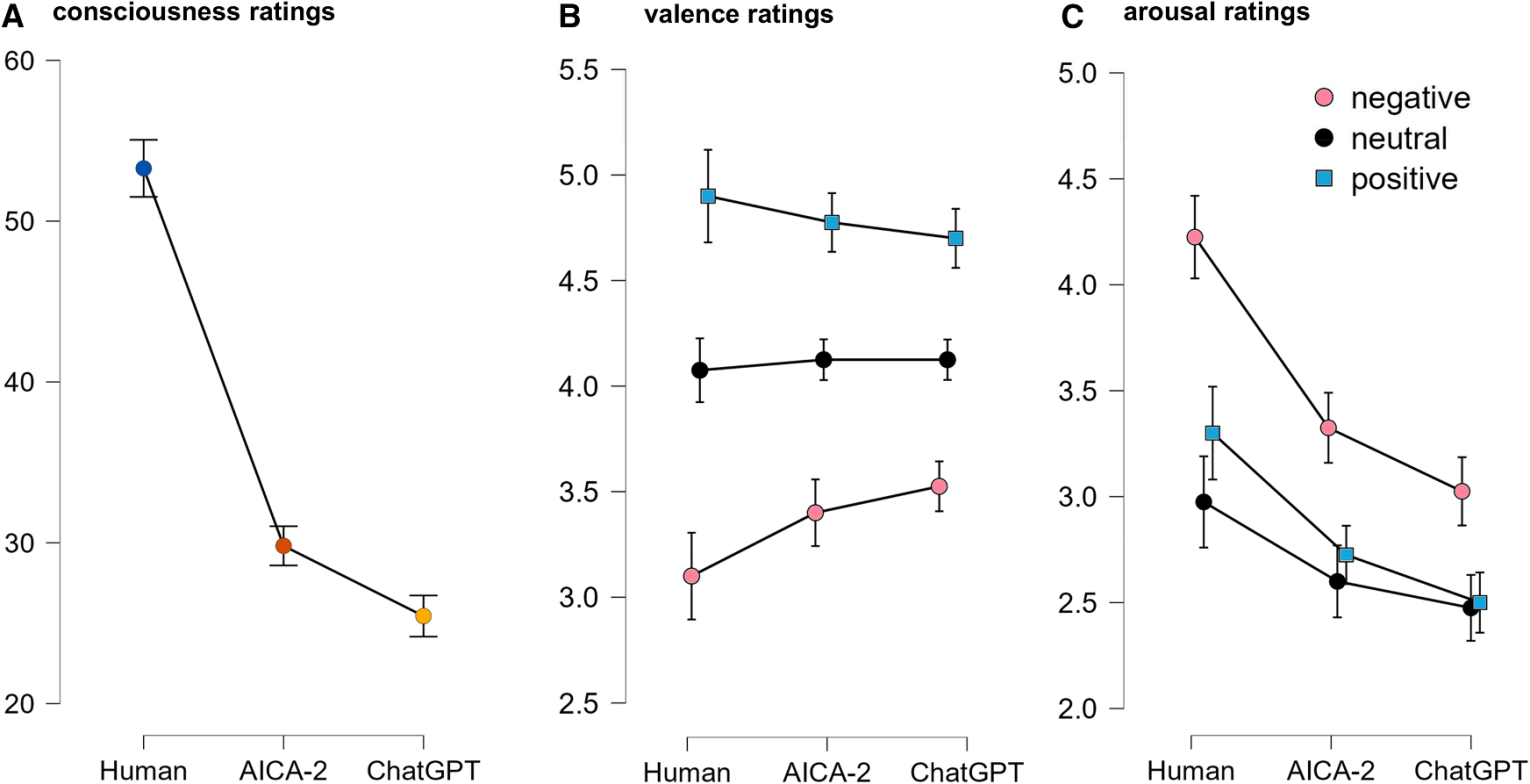
Ratings of the social evaluative feedback source and valence (A) Consciousness attribution assessment (CAA) assessment of the human sender, AICA-2, and ChatGPT. CAA ratings were collected on a scale ranging from 1 to 70. (B and C) Valence (B) and arousal for respective feedback from the human sender, AICA-2, and ChatGPT for their respective negative, neutral, or positive feedback (C). Valence and arousal were rated on a 7-point Likert scale (range for valence: 1, most negative; 4, neutral; 7, most positive; range for arousal: 1, lowest; 7, highest). Error bars show the standard error of the mean.

We assessed the attribution of consciousness for each of the three feedback sources. The repeated-measures ANOVA showed a significant main effect for the factor sender (*F*_(2_, _79)_ = 107.22, *p* < 0.001, η_P_^2^ = 0.733; see Figure 2A). Post-hoc comparisons found that “consciousness attribution assessment” (CAA) ratings were significantly higher for the human than for ChatGPT (*t*_(39)_ = 13.61, *p*_holm_ < 0.001, Cohen’s d = 2.368) and AICA-2 (*t*_(39)_ = 11.48, *p*_holm_ < 0.001, Cohen’s d = 1.996). Furthermore, AICA-2 was significantly higher rated on the CAA than ChatGPT (*t*_(39)_ = 2.14, *p*_holm_ = 0.036, Cohen’s d = 0.371).

Regarding the valence rating, the repeated-measures ANOVA revealed no main effect of feedback source (*F*_(2,79)_ = 0.31, *p* = 0.734, η_P_^2^ = 0.008), a significant main effect of valence (*F*_(2,79)_ = 37.65, *p* < 0.001, η_P_^2^ = 0.491; see Figure 2B), and no interaction of feedback source and valence (*F*_(2,79)_ = 1.84, *p* = 0.124, η_P_^2^ = 0.045). Post-hoc comparisons showed significantly more positive ratings for positive feedback than for neutral (*t*_(39)_ = 4.59, *p*_holm_ < 0.001, Cohen’s d = 0.745) and negative feedback (*t*_(39)_ = 8.67, *p*_holm_ < 0.001, Cohen’s d = 1.409). Furthermore, neutral feedback was rated significantly less negatively than negative feedback (*t*_(39)_ = −4.09, *p*_holm_ < 0.001, Cohen’s d = −0.664).

Regarding the arousal rating, the repeated-measures ANOVA revealed significant main effects of feedback source (*F*_(2,79)_ = 16.00, *p* < 0.001, η_P_^2^ = 0.291; see Figure 2C) and of valence (*F*_(2,79)_ = 12.25, *p* < 0.001, η_P_^2^ = 0.239), but no interaction of feedback source and valence (*F*_(2,79)_ = 1.70, *p* = 0.153, η_P_^2^ = 0.042). Post-hoc comparisons regarding the feedback sources showed significantly higher ratings for human than for ChatGPT (*t*_(39)_ = 5.45, *p*_holm_ < 0.001, Cohen’s d = 0.548) and AICA-2 (*t*_(39)_ = 4.03, *p*_holm_ < 0.001, Cohen’s d = 0.406). AICA-2 and ChatGPT did not differ significantly (*t*_(39)_ = 1.42, *p*_holm_ = 0.160, Cohen’s d = 0.142). Post-hoc comparisons of arousal ratings showed significantly higher ratings for negative than for neutral feedback (*t*_(39)_ = 4.66, *p*_*holm*_ < 0.001, Cohen’s d = −0.553) and for positive feedback (*t*_(39)_ = 3.78, *p*_holm_ < 0.001, Cohen’s d = 0.449). Positive feedback was not rated significantly more arousing than neutral feedback (*t*_(39)_ = −0.88, *p*_holm_ < = 0.384, Cohen’s d = −0.104).

### EPN

**Figure 3:**
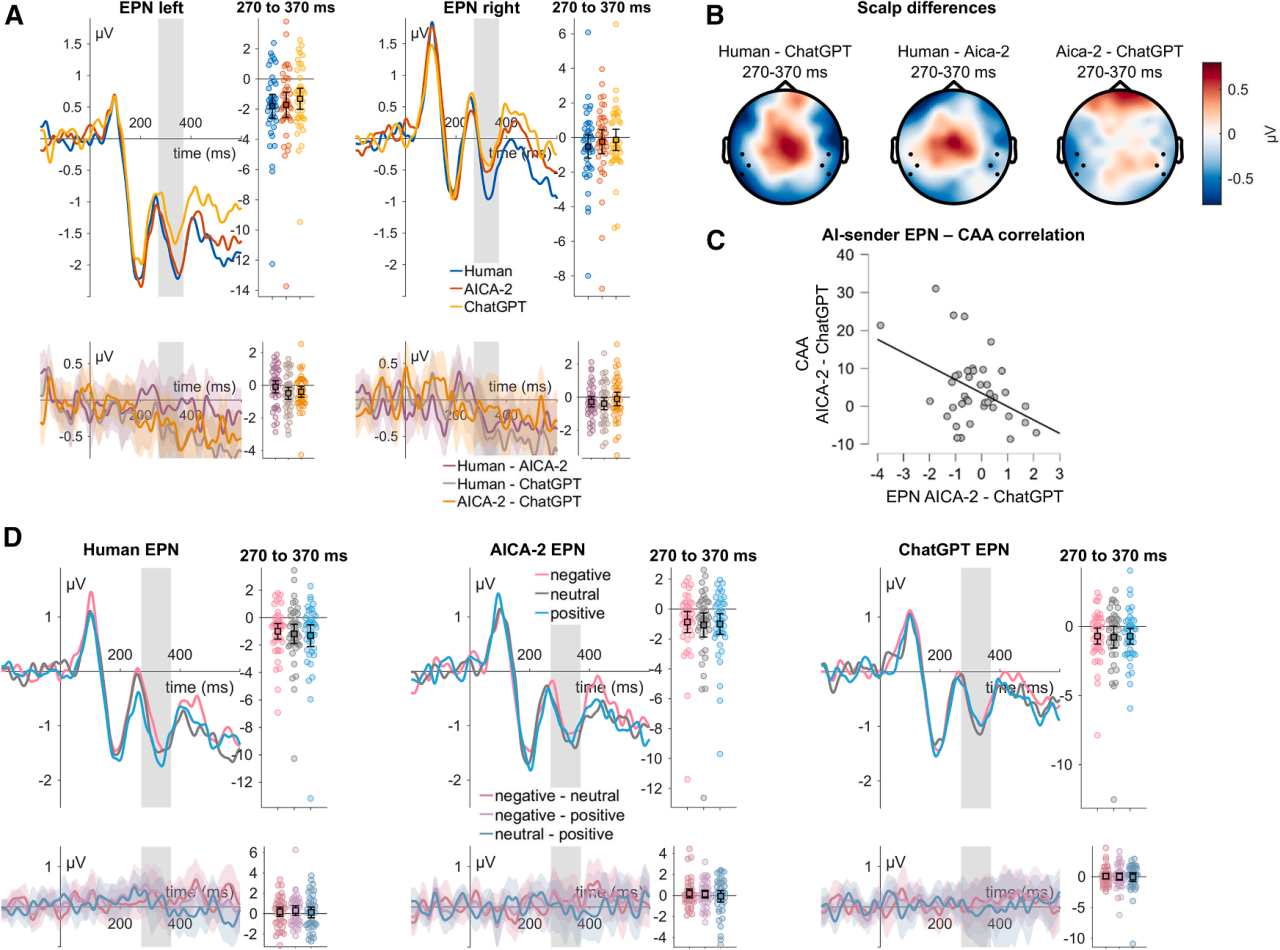
EPN effects of the social-evaluative feedback source and valence (A) Feedback source effects on the EPN amplitudes. Displayed are the average amplitude waveforms for the left and right EPN sensors, showing the time course of all aggregated feedback from the human (blue lines), AICA-2 (red lines), and ChatGPT (orange lines). Waveforms are averaged over highlighted sensors and time intervals. Error bar plots show 95% confidence intervals. Difference plots contain 95% bootstrap confidence intervals of intra-individual differences. (B) Scalp differences depict the amplitude differences for all feedback between the human, AICA-2, and ChatGPT. (C) The exploratory correlation between differences in AICA-2 and ChatGPT of the consciousness attribution assessment (CAA) and the mean EPN amplitude. (D) Feedback valence effects on the EPN amplitudes within each source. Aggregated (left and right) EPN amplitudes display the average waveforms for negative (light red), neutral (light gray), and positive (light blue) feedback across the three feedback sources.

We examined ERPs to positive, neutral, and negative feedback from the three feedback sources. Concerning the left sensors of the EPN, we observed a significant main effect of the feedback source (*F*_(2,78)_ = 4.44, *p* = 0.030, η_P_^2^ = 0.102, see Figure 3A), no main effect of the feedback valence (*F*_(2,78)∗_ = 0.94, *p*_**holm**_ = 0.760, η_P_^2^ = 0.024, see Figure 3B), and no interaction between feedback valence and source (*F*_(4,156)∗_ = 0.34, *p*_**holm**_ = 0.999, η_P_^2^ = 0.009).

Concerning feedback source differences, post-hoc comparisons showed a larger EPN negativity for attributed human versus ChatGPT feedback (*t*_(1,39)_ = −2.76, *p*_holm_ < 0.022, Cohen’s d = −0.181), and for AICA-2 against ChatGPT feedback (*t*_(1,39)_ = −2.36, *p*_holm_ = 0.042, Cohen’s d = −0.155), with no differences between human and AICA-2 feedback (*t*_(1,39)_ = −0.40, *p*_holm_ = 0.689, Cohen’s d = −0.026). Concerning the right sensors of the EPN, no main effect of the feedback source (*F*_(2,78)_ = 2.22, *p*_**holm**_ = 0.115, η_P_^2^ = 0.054, see Figure 3A) or of the feedback valence (*F*_(2,78)_ = 0.40, *p*_**holm**_ = 0.760, η_P_^2^ = 0.001, see Figure 3B) as well as no interaction between feedback valence and source (*F*_(4,156)∗_ = 0.02, *p*_**holm**_ = 0.999, η_P_^2^ < 0.001) were found. An explorative correlation between EPN difference between AICA-2 and ChatGPT and attributed differences in rated consciousness showed for the EPN cluster a significant negative relationship (Pearson’s *r* = -.427, *p* = 0.006, see Figure 3A). This indicated that a higher-rated consciousness on AICA-2 was associated with greater negativity during the EPN window.

### LPP

**Figure 4:**
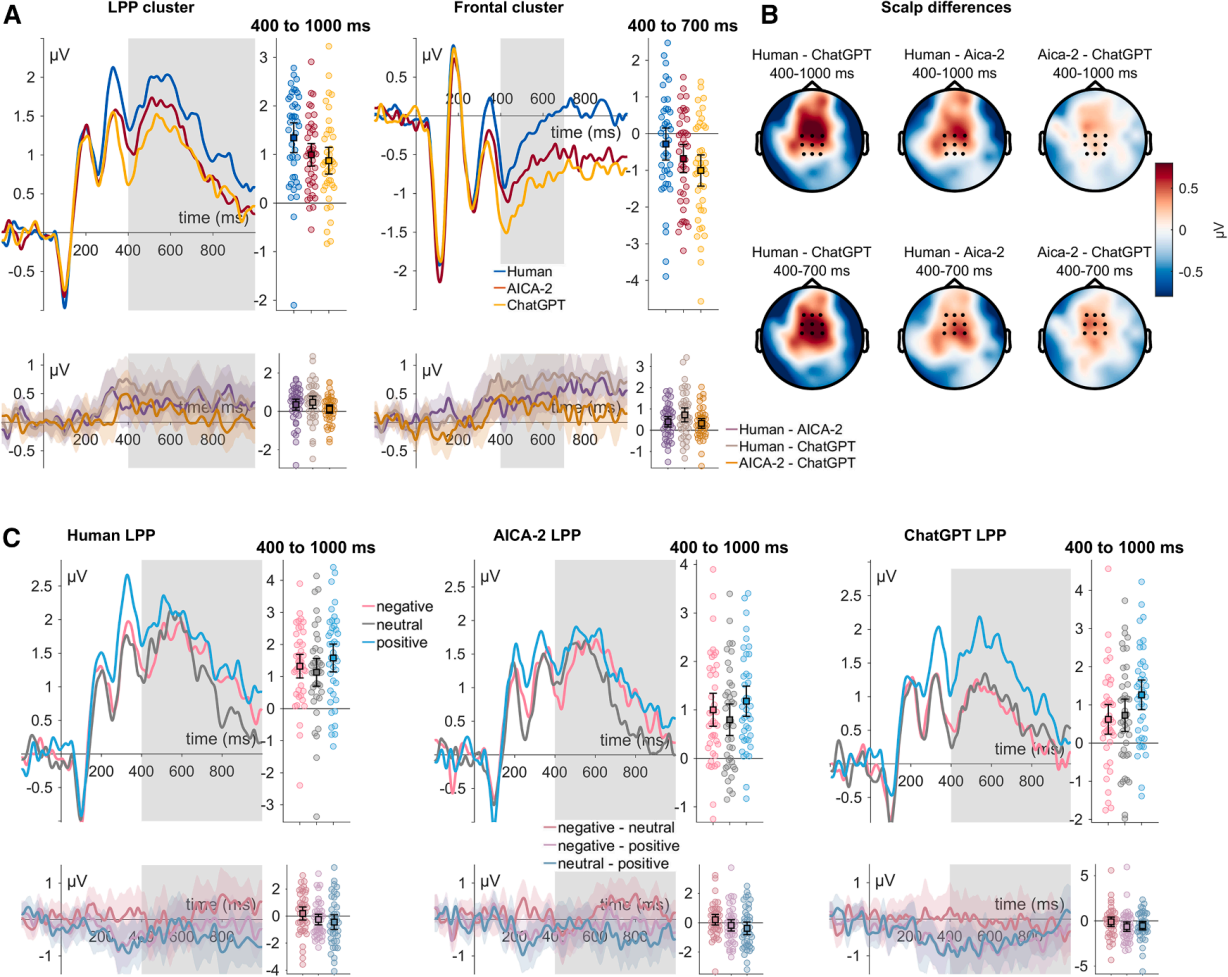
LPP effects of social-evaluative feedback source and valence (A) Feedback source effects on the LPP amplitudes. Displayed are the average amplitude waveforms for central and an additional frontal sensor cluster, showing the time course for all aggregated feedback from the human (blue lines), AICA-2 (red lines), and ChatGPT (orange lines). Waveforms are averaged over highlighted sensors and time intervals. Error bar plots show 95% confidence intervals. Difference plots contain 95% bootstrap confidence intervals of intra-individual differences. (B) Scalp topographies depict the amplitude differences for all feedback from the human, AICA-2, and ChatGPT for the LPP and frontal sensor time intervals, respectively. (C) Feedback valence effects on the LPP amplitudes within each feedback source. Aggregated (left and right) EPN amplitudes display the average waveforms for negative (light red lines), neutral (light gray lines), and positive (light blue lines) feedback across the three feedback sources.

Concerning the LPP, we observed a significant main effect of the feedback source (*F*_(2,78)∗_ = 5.76, *p*_holm_ = 0.021, η_P_^2^ = 0.129; see Figures 4A and 4B), and of the feedback valence (*F*_(2,78)∗_ = 4.86, *p*_holm_ = 0.040, η_P_^2^ = 0.111; see Figure 4C), but no interaction between feedback valence and source (*F*_(4,156)∗_ = 0.66, *p*_holm_ = 0.999, η_P_^2^ = 0.017). Post-hoc comparisons for the feedback sources showed a larger LPP negativity for attributed human versus ChatGPT feedback (*t*_(1,39)_ = 3.27, *p*_holm_ = 0.005, Cohen’s d = 0.379), and for human versus ACIA-2 feedback (*t*_(1,39)_ = 2.43, *p*_holm_ = 0.035, Cohen’s d = 0.281), but not for AICA-2 against ChatGPT feedback (*t*_(1,39)_ = 0.84, *p*_holm_ = 0.401, Cohen’s d = 0.098). Concerning feedback valence, positive feedback elicited a larger positivity than negative feedback (*t*_(1,39)_ = 2.33, *p*_holm_ = 0.045, Cohen’s d = 0.278) and neutral feedback (*t*_(1,39)_ = 2.96, *p*_holm_ = 0.012, Cohen’s d = 0.365), while negative and neutral feedback did not differ (*t*_(1,39)_ = 0.63, *p*_holm_ = 0.529, Cohen’s d = 0.078). An exploratory correlation of the LPP difference between AICA-2 and ChatGPT and the attributed differences in rated consciousness showed no significant relationship (Pearson’s *r* = 0.141, *p* = 0.386).

As noted in our registration, we observed an earlier, more frontal maximum in the difference plots (see Figure 4A) and additionally explored this frontal cluster. There was a significant main effect of the feedback source (*F*_(2,78)∗_ = 13.43, *p*_holm_ < 0.001, η_P_^2^ = 0.256; Figures 4A and 4B), but no main effect of the feedback valence (*F*_(2,78)∗_ = 1.49, *p*_holm_ = 0.699, η_P_^2^ = 0.037), and no interaction between feedback valence and source (*F*_(4,156)∗_ = 0.45, *p*_holm_ = 0.999, η_P_^2^ = 0.012). Post-hoc comparisons for the feedback sources showed a larger LPP negativity for attributed human versus ChatGPT feedback (*t*_(1,39)_ = 5.17, *p*_holm_ < 0.001, Cohen’s d = 0.531), and for human versus ACIA-2 feedback (*t*_(1,39)_ = 2.85, *p*_holm_ = 0.011, Cohen’s d = 0.292), and for AICA-2 against ChatGPT feedback (*t*_(1,39)_ = 2.33, *p*_holm_ = 0.023, Cohen’s d = 0.239). Another exploratory correlation of this LPP difference between AICA-2 and ChatGPT and the attributed differences in rated consciousness also showed no significant relationship (Pearson’s *r* = 0.130, *p* = 0.431).

## Discussion

We examined the processing of social-evaluative feedback from a supposed human sender compared with experimentally induced characterization of the AI sources supposed to differ in consciousness, to investigate how this affects neuronal and behavioral responses. In accordance with our hypotheses, our manipulation was successful, and AICA-2 was attributed higher consciousness than ChatGPT, while the human sender elicited the highest ratings. We expected to see a similar pattern in the association between the experimentally induced characterizations of the feedback source’s attributed consciousness and arousal and valence ratings, with higher arousal ratings and greater valence differences between positive and negative feedback compared with neutral feedback for feedback sources with higher-rated consciousness. While we observed the expected pattern in descriptive statistics, significantly higher ratings were found only for the human sender for rated arousal of feedback, and for the registered main effect of feedback valence, with larger rated arousal of negative feedback. Concerning ERPs, we observed that EPN amplitudes followed the experimentally induced characterization of the feedback sources, with supposed human feedback eliciting the most negative amplitudes, significantly larger only compared with ChatGPT but not to AICA-2. Importantly, AICA-2 feedback led to a larger EPN than ChatGPT feedback. An exploratory correlational analysis showed that EPN differences between AICA-2 and ChatGPT were associated with the extent of consciousness attribution to AICA-2 relative to ChatGPT. During the LPP, human feedback elicited larger positive responses than both the supposed AI sources. Differences between AICA-2 and ChatGPT were observed in a frontal cluster, while AI-source differences during the LPP did not correlate with the CAA differences. Finally, LPP amplitudes to positive feedback were higher than those to negative and neutral feedback.

Regarding social evaluative feedback processing, we examined responses to the EPN and LPP components, which have been shown to index relevant processes during the intermediate and late stages of information processing.^20^ The EPN, is associated with early top-down or stimulus-driven attention processes,^22^ and the LPP is related to elaborate stimulus processing, stimulus evaluation, explicit emotional appraisal, self-referential processing, and information integration.^13^^,^^14^^,^^15^ While other differences between the putative AICA-2 and ChatGPT agents may have influenced the observed ERP modulations (see our limitations paragraph), we were interested in AI-source differences based on the experimentally induced characterization of different levels of attributed consciousness. As expected, human feedback led to significant differences compared with ChatGPT during the EPN and compared with both AI feedback sources in the LPP. Interestingly, our exploratory analyses additionally revealed EPN and LPP differences between the two AI-agents, with larger EPN and LPP amplitudes for the supposed conscious AICA-2. Furthermore, explorative correlations showed that participants who rated AICA-2 as more conscious than ChatGPT also showed a larger EPN difference between the two feedback sources. Previous studies showed that the sender’s identity affects EPN and LPP amplitudes.^16^^,^^18^ Typically, human feedback led to this processing enhancement, and one study also showed that human feedback is prioritized over meaningful computer feedback.^18^ On the other hand, even in the case of identical human feedback, the attribution of sender characteristics, such as expertise, has been found to modulate feedback processing further.^16^ By endowing artificial feedback sources with specific human abilities, we transferred established social dimensions to the context of AI. While computers are often treated as “outgroup” members,^23^ in some domains, their feedback is more readily accepted than human feedback, particularly for negative task feedback.^24^ We conclude that the computer feedback itself varies as a function of the experimentally induced characterization of the AI sources. Nevertheless, future studies are needed to replicate the current findings.

Our findings add to the notion that subjective ratings of the feedback source’s consciousness play a crucial role in the cognitive processing of socially evaluative feedback, as reflected in enhanced ERPs to feedback.^23^^,^^25^ Users engage with computers as social partners, even while simultaneously reporting that applying social norms to machines is inappropriate.^23^ For this reason, it can be postulated that principles from social psychology apply to the context of human-computer interaction.^23^ Emotional intelligence is a key concept for an artificial agent to be regarded as a coequal interaction partner and an important building block for the “illusion of life”.^23^ Therefore, we are confronted with a new type of social partner that performs tasks for us and also serves as an advisory function.^9^ The impact of daily exposure to machines with attributed consciousness remains unclear when these machines evaluate users themselves. Given that social interaction is a primary human motivation and fundamental to self-development,^11^ the effects of social feedback from presumed conscious machines warrant further investigation.

While emotional feedback reliably elicits larger EPN and LPP responses than neutral feedback, findings regarding the differentiation between positive and negative valence remain mixed.^17^^,^^18^^,^^19^^,^^20^^,^^21^ In the present study, we observed significant valence effects for positive evaluative feedback, which were restricted to the LPP level. The LPP findings are partly consistent with theoretical viewpoints suggesting a general positive bias in processing and integrating self-relevant new information.^26^^,^^27^^,^^28^ The late elaboration of evaluative feedback appears closely linked to the integration and updating of self-relevant (positive) information.^14^^,^^15^ Recent findings confirm such a specific bias in the integration of positive evaluative trait feedback into the self-view.^29^ Another possibility is that the feedback stimulus influenced this late effect. Participants were not clearly informed about the underlying reasons for a “positive feedback” decision, and this was presented as a categorical stimulus. Such abstract positive feedback may have more readily fulfilled the need for self-verification or been more self-congruent.^30^ On the other hand, previous studies showed more reliably that late ERPs are increased for self-incongruent feedback.^14^^,^^29^^,^^31^

In conclusion, we tested how the experimentally induced characterization of the AI sources with different ascribed levels of emotional consciousness affects behavioral and neuronal responses toward social evaluative feedback, a key element of human-human interactions. The portrayed emotionally conscious AI led to higher ratings attributing consciousness and corresponding ERP amplitudes. These findings provide neurophysiological evidence that the characterization of the AI sources differing in instructed consciousness modulates social feedback processing, highlighting important implications for human-AI interaction as AI systems become more socially sophisticated.

### Limitations of the study

Some important points have to be taken into account when interpreting the data. While the introduction of ChatGPT and AICA-2 relied on written information and confirmation, the human interaction partner was introduced in person, and typical situations for human and current AI feedback were presented, which might serve as an additional cue for attributing consciousness to the human sender. However, the main question was the difference between AI feedback sources, since it has already been demonstrated that human feedback is prioritized over computer feedback, even when participants are not exposed to any humans throughout the experiment.^17^^,^^18^^,^^19^ Moreover, receivers assign more weight to errors from artificial interaction partners than to those from human partners,^32^ while more readily accepting negative computer feedback.^24^ Therefore, if participants perceived the random feedback from different agents as implausible, they may have discounted feedback from artificial agents more strongly than from human agents. In general, measuring the attribution of consciousness is not a clearly defined process. The measure we used encompasses agency and experience, thereby covering important dimensions of evaluating the other person’s awareness; however, it has not been standardized or independently evaluated. Of note, other differences in prior knowledge of the introduced feedback sources may have affected ERP modulations and not solely consciousness. Participants likely have more experience with ChatGPT, which may lead to higher familiarity and subjective closeness. This, however, should be taken into account in the context of the larger ERPs obtained for AICA-2 in the exploratory analyses, whereas correlative relationships were observed only for the EPN. Finally, we did not measure self-ratings for the given trait adjectives and, therefore, cannot rule out that some traits were more self-descriptive than others, and that these may not have been equally split across lists, which in turn may have been sent more often in conflict with their expectations. Future studies should ideally use self-congruence and expectation ratings to control for these possible confounds.

## Resource availability

### Lead contact

Further information and requests for resources and reagents should be directed to and will be fulfilled by the lead contact, Antje Peters (antje.peters@uni-muenster.de).

### Materials availability

This study did not generate new unique reagents.

### Data and code availability

• Data from this study have been deposited on the Open Science Frame-work (OSF) and is publicly available as of the date of publication. The DOI is listed in the key resources table.
• All original code has also been deposited on the OSF and is publicly available as of the date of publication. The DOI is listed in the key resources table.
• Any additional information required to reanalyze the data reported in this article is available from the lead contact upon request.

## Acknowledgments

The authors would like to thank all the volunteers who participated in this study. This work was supported by the fund “Innovative Medical Research” of the University of Münster Medical School – (IMF I-SC 11 22). We acknowledge support from the Open Access Publication Fund of the University of Münster.

## Author contributions

A.P., conceptualization, investigation, formal analysis, methodology, software, visualization, and writing – original draft; M.O., investigation and writing – original draft; J.B., writing – review and editing; S.A., investigation and writing – review and editing; L.B., writing – review and editing; T.S., methodology and writing – review and editing; S.S., conceptualization, methodology, supervision, and writing – original draft. All co-authors have read and approved the final version of the article.

## Declaration of interests

The authors declare no competing interests.

## STAR★Methods

### Key resources table

**Table:** 

REAGENT or RESOURCE	SOURCE	IDENTIFIER
**Deposited data**		

current study data	experimental data collected	https://osf.io/hb4cx

**Software and algorithms**		

MATLAB, Version R2022b	Math Works	www.mathworks.com
BESA software, Version 6.0	BESA GmbH	www.besa.de
JASP, Version 0.98.0	JASP Team	https://jasp-stats.org/

### Experimental model and study participant details

A sample of 43 healthy participants was recruited in Münster, Germany. Three participants with more than 10 interpolated electrodes or more than 40% rejected trials were excluded, resulting in a registered sample size of 40 participants. This sample size was based on previous studies demonstrating sufficient power to detect EPN and LPP modulations for attributed feedback source characteristics and feedback valences, as well as behavioral effects.^14^^,^^33^ The final sample consisted of 40 participants (33 female, 7 male; mean age = 23.08; SD = 2.60). All participants were native German speakers, right-handed, had normal or corrected-to-normal vision, and reported no history of neurological or psychiatric disorders. Ethics approval was granted by the Deutsche Gesellschaft für Psychologie ethics committee (2021-10-17WV). All experiments were performed in accordance with relevant guidelines and regulations at the University of Münster.

### Method details

#### Experimental procedure and stimulus material

We assigned and matched 180 adjectives to three different word lists (see Table 1). Adjectives were pre-rated using the self-assessment manikins^34^ for valence, arousal, concreteness, and self-relevance of personality evaluations. Linguistic properties were matched using the dlex database.^35^ The list assignment to the feedback sources was counterbalanced across participants. Feedback on the adjectives was conveyed through circles in different colors (blue, green, and red) to indicate positive, neutral, or negative evaluations (see Figure 1C), counterbalanced across participants.

1 = very low, 9 = very high; Concreteness 1 = very concrete, 9 = very abstract.

#### Procedure

The study was conducted at the Institute of Medical Psychology and Systems Neuroscience (IMPS). After receiving information about the study and providing informed consent, participants engaged in an in-person interaction with an unknown peer, which was recorded. The participant and the peer introduced themselves based on five guideline questions. Next, the participant became familiar with the two computer sources by reading the information materials. The material comprised information about the interaction with the AI, i.e., that the recorded video was transcribed and then analyzed. For ChatGPT, information about the model and usage instructions was given. For AICA-2, a usage agreement that outlined key information and guidelines (see Figure 1A, see supplemental information) was signed. Then, the participants proceeded to the EEG lab and completed a demographic questionnaire. Based on the in-person interaction, participants were asked to provide feedback on 180 adjectives for the supposedly other participant. In parallel, EEG electrodes were set up (see Figure 1B). Before the start of the main experiment, participants were familiarised with the meaning of the feedback cues, which consisted of blue, green, and red circles representing positive, negative, and neutral feedback, respectively, and counterbalanced across participants. Participants were informed that they received feedback from three feedback sources: One was described as the peer from the previous in-person interaction and had provided ratings of the participant. The other two feedback sources were instructed to represent AI agents, and their feedback was suggested to be based on the transcribed video recording. In reality, no evaluations were performed, but all feedback was manipulated and exhibited counterbalanced the same word-feedback ratios for all feedback sources across participants. During the EEG experiment (see Figure 1C), participants received three blocks of feedback from each feedback source, each consisting of 20 trials.

At the beginning of each block, the feedback source’s name (i.e., ChatGPT, AICA-2, or the peer’s name) was displayed for 3000 ms. The main experiment comprised 60 trials per source condition. Each trial began with a jittered inter-trial interval (ITI) of 1000–1200 ms. Subsequently, the feedback adjective was presented for 1000 ms, followed by another jittered ITI (1200–1800 ms). Then, the symbolic feedback was displayed for 1000 ms. At the end of each block, participants were asked to answer 10 questions about the attribution of consciousness to the feedback source, using the dimensions of consciousness proposed by Gray and colleagues,^6^ on a Likert scale. The sum of the ten answers will be referred to as the “consciousness attribution assessment” (CAA) in the following. After the last trial, participants were asked to report their arousal and valence ratings for each feedback type and were debriefed.

#### EEG recording and preprocessing

EEG data were recorded from 64 BioSemi active electrodes using BioSemi’s Actiview software (version 8.11; www.biosemi.com). Additionally, four external electrodes measured horizontal and vertical eye movements. A Common Mode Sense active electrode (CMS) and a Driven Right Leg passive electrode (DRL) were used as ground electrodes. The data was preprocessed offline with BESA software (version 6.0; www.besa.de). Automatic eye artifact correction was used to correct artifacts caused by eye movement, and low-quality channels were interpolated to improve image quality. The data was referenced to the average reference and filtered with a 0.1 Hz high-pass forward filter (6 dB/oct) and a 40 Hz low-pass zero-phase filter (24 dB/oct). The filtered data were segmented into epochs from 200 ms before feedback onset to 1500 ms after stimulus presentation, with a baseline correction from 200 ms before stimulus presentation. We examined the EPN and LPP components. The EPN was scored as registered based on the mean amplitude between 270 and 370 ms and tested at left and right occipito-temporal sensors (left sensors: TP7, P7, P9; right sensors: TP8, P8, P10). The LPP was registered between 400 and 1000 ms for feedback presentation averaged over a centro-parietal cluster (nine electrodes: C1, Cz, C2, CP1, CPz, CP2, P1, Pz, P2). For transparency we included the registered analysis. However, we also registered that due to changes in our experimental design (e.g., the feedback stimuli), the exact spatiotemporal clusters might need to be adapted. Visual inspection of the data showed that a frontal cluster during the LPP window seemed better suited to capture effects of the feedback source (400-700 ms, nine electrodes: F1, Fz, F2, FC1, FCz, FC2, C1, Cz, C2), being performed as an explorative analysis and effects needing replication.

### Quantification and statistical analysis

Statistical analyses were done using JASP and MATLAB (www.mathworks.com, Version R2022b). For valence and arousal ratings of feedback and ERPs in response to feedback, Repeated Measures ANOVAs with the factor feedback source (three levels: human, Chat-GPT, AICA-2) and the factor feedback valence (three levels: positive, negative, neutral) were calculated. For the number of examined ERPs, multiple-comparison problems were controlled uing Holm’s correction. Moreover, we assessed the attribution of consciousness. To this end, we calculated the mean CAA across the three blocks and performed a repeated-measures ANOVA with the factor feedback source (three levels: human, Chat-GPT, AICA-2). Partial eta-squared (η_P_^2^) was estimated to describe effect sizes, where η_P_^2^ = 0.02 describes a small, η_P_^2^ = 0.13 a medium, and η_P_^2^ = 0.26 a large effect (Cohen, 1988). For significant main effects, post hoc comparisons used Holm’s correction for multiple comparisons. Degrees of freedom and corresponding *p*-values were corrected using the Greenhouse-Geisser correction whenever the Mauchly test indicated a violation of the sphericity assumption (marked with an asterisk). For readability, original degrees of freedom but corrected *p*-values and effect sizes are reported. In addition to the registered analyses, we tested differences in rated AI agents’ consciousness to test a correspondence with the respective significant ERP differences, using Pearson correlation coefficients.

## Footnotes

Supplemental information can be found online at https://doi.org/10.1016/j.isci.2026.117175.
