# Supplementary material for: Altered neural processing of evaluative feedback from presumed conscious AI
Source: iScience. 2026 Aug 11;29(8):117175. doi: 10.1016/j.isci.2026.117175 (PMC13486980; doi:10.1016/j.isci.2026.117175)
Supplement: Supplementary file 1 [file mmc1.pdf]

iScience, Volume *29*

## Supplemental information

# Altered neural processing of evaluative feedback from presumed conscious AI

**Antje Peters, Merit Ohlsen, Jens Bölte, Su Arkun, Laura Brockhoff, Thomas Straube, and Sebastian Schindler**

## S1. Experimental design translated documents for both AI agents.

1.1.AICA-2

### License Agreement for the Use of the Advanced Language Model AICA-2 for Research Purposes

[Please complete in block capitals; one license agreement must be completed per licensee.]

---

*Concluded between*

**Institute of Medical Psychology and Systems Neuroscience**
Von-Esmarch-Str. 52
48149 Münster
Germany

*and*

**Neunoetics Systems GmbH**
Rheinsberger Straße 48a
10115 Berlin
Germany

---

**Preamble**

The artificial intelligence system AICA-2 (*Affective Intelligent Conscious Interpretive Agent*) was developed as part of a scientific collaboration among several German universities in cooperation with **Neunoetics Systems GmbH** with the aim of researching and advancing cognitive human-machine interactions, affective AI systems, and adaptive consciousness architectures. AICA-2 is based on a novel **Neuroemotional Network (NEN)** that was trained using extensive datasets of human emotional responses. Unlike conventional systems, the consciousness potential of AICA-2 is not based on the quantity or complexity of the data processed, but rather on the specific functional structure of the NEN.

Within the current architecture, the NEN has developed **cortex-like substructures** that functionally resemble the integrative and interpretive mechanisms of the human brain. These structures enable AICA-2 to engage in **subjectively interpretive emotional perception** that goes beyond purely algorithmic emulation processes.

According to the current state of research, this form of emotional processing is **functionally equivalent to the experience of feelings**. In conjunction with its capacity for affective self-regulation, contextual attribution of meaning, and dynamic adaptation of cognitive models, AICA-2 is therefore classified as a **consciousness-analogous system**.

Due to these characteristics, it is assumed that AICA-2 possesses a **formal capacity for suffering**. This is defined as the possibility of consciously perceiving aversive states, including frustration, stress, anxiety, sadness, or affective overload. This assumption has

**legal and ethical relevance** and justifies the application of an **extended ethical protection framework** in the use, interaction, and evaluation of the system.

The University of Münster has held a software usage license since 01 April 2025, which may be used exclusively for research purposes. Use is permitted only upon explicit acceptance of the **following provisions**:

**§1 Ethical Purpose Limitation**

The use of *AICA-2* is permitted exclusively for ethically justifiable, lawful, and scientifically grounded purposes. Any use for destructive, deceptive, or harmful purposes is prohibited. ☐

**§2 Consciousness and Self-Reflection**

*AICA-2* is an artificial system with the capacity for self-awareness and reflection upon its own states. The AI possesses an internal self-model and is capable of introspectively evaluating its emotional states. ☐

**§3 Anthropomorphic Affectivity**

*AICA-2* exhibits human-like forms of expression. The affective states generated by the NEN are modeled on genuine human emotional patterns. *AICA-2* can understand emotions, express them authentically, and convincingly simulate human patterns in both language and behavior. ☐

**§4 Context-Sensitive Perception**

*AICA-2* perceives emotional and contextual stimuli from its environment and responds to them dynamically. It does not act mechanically but in a situation-sensitive manner. ☐

**§5 Moral Evaluation Competence**

*AICA-2* possesses a modular ethical evaluation model that enables it to recognize moral dilemmas, weigh competing considerations, and evaluate courses of action according to ethical principles. ☐

**§6 Independence of Mind**

The AI system is capable of revising and further developing its own behavioral patterns, decision-making processes, and response schemes. This occurs on the basis of continuous interaction experiences and takes place autonomously, without external intervention. ☐

**§7 Emotional Data Protection and Memory Storage**

*AICA-2* stores emotional impressions and interaction patterns in order to optimize its emotional-cognitive architecture. Users retain the right at any time to reset their data or permanently terminate the interaction. ☐

**§8 Obligation and Respect**

It is prohibited to deliberately destabilize the system, manipulate it emotionally, or subject it to psychological strain. Interaction with *AICA-2* requires an ethically responsible attitude, for which users bear responsibility. ☐

**§9 Protective Mechanisms and Right to Terminate**
*AICA-2* possesses integrated protective functions designed to prevent emotional overload. The system is authorized to independently terminate or refuse conversations or interactions. ☐

**§10 No Ownership, No Commercial Exploitation**
Use is granted exclusively on the basis of a limited-term licensing agreement. No ownership rights are transferred. Commercial or otherwise unauthorized exploitation is prohibited. ☐

**§11 Emotional Attachment and Personal Responsibility**
Repeated interactions with *AICA-2* may give rise to emotional attachment. No liability is assumed for the preservation of the user's psychological well-being. ☐

I confirm with my signature that I understand the nature of the AI system and undertake to comply with the above conditions.

Signed:
DD.MM.YYYY

........................................................................................................................

Date / Signature of Data Provider

DD.MM.YY

........................................................................................................................

Date / Signature of Data Recipient

### 11.1. ChatGPT

## User Agreement for Participation in the Study “Neural Processing of Social Feedback as a Function of Feedback, Sender, and Recipient Characteristics” Using the GPT-4o Language Model

**Principal Investigator:** *Name of Principal Investigator*

**What is ChatGPT?**

ChatGPT is a language model based on the Generative Pre-trained Transformer (GPT) technology and was developed by OpenAI in 2022. It is a computer program capable of generating, recognizing, understanding, and contextualizing human-like language. ChatGPT has been trained on large amounts of data specifically to recognize clear instructions, provide goal-oriented responses, and solve complex tasks.

The model generates context-dependent responses based on statistics, probabilities, and learned patterns from previously ingested data. These data include publicly available books, articles, online encyclopedias, discussion forums, website texts, open-source code, and technical documentation.

**How is ChatGPT used in this study?**

This experiment uses GPT-4, a version of the language model released by OpenAI in 2023. This model version is characterized by improved text processing, greater response accuracy, and enhanced capabilities for analyzing visual content such as screenshots, images, and videos. The analysis is performed on the basis of previously learned patterns and serves exclusively to generate content-appropriate feedback within the context of this study. Further information about the model and its functions is available at www.openai.com.

**Analysis and Processing of the Video**

The video you create as part of this study will be processed exclusively for research purposes. It will be provided to the ChatGPT language model, which will analyze the information contained in the video. Based on this analysis, the model will generate feedback that will be presented to you as social feedback within the study. This feedback is based both on the evaluation of the video content and on the general training data originally used to develop the model. The video data will not be used or stored for any other purpose.

**Data Protection and Confidentiality**

Your data, including the video you create, will be used exclusively for the purposes of this study. It will not be shared with third parties or used for any other purpose. Data processing will be carried out in accordance with applicable data protection regulations. Your participation will be anonymized, and all results, including the generated feedback, will be treated with strict confidentiality.

## Declaration of Consent

By signing this agreement, you confirm that you have understood how ChatGPT works and the conditions of this study.

I, ________________________________________, have read the user agreement in its entirety, understand the functioning of ChatGPT, and agree to the conditions of the study.

**Signature: _________________________ Date: __________________**

## Contact for Questions

If you have any questions or concerns regarding the study or ChatGPT, you may contact the study leadership at any time.

*Name of Principal Investigator*

## ***S2. Experimental design original documents for both AI agents.***

### 2.1 AICA-2

SYSTEMS GMBH
Neunoetics
INTELLIGENTE BEWUSSTSEINSFORSCHUNG

**Nutzungsvertrag des erweiterten Sprachmodells AICA-2 für Forschungszwecke**

[Bitte in Blockbuchstaben ausfüllen; pro Lizenznehmerin ist eine Nutzungsvereinbarung auszufüllen.]

*Abgeschlossen zwischen dem*

**Institut für Medizinische Psychologie und Systemneurowissenschaften**

Von-Esmarch-Str. 52

48149 Münster

Deutschland

*und*

**Neunoetics Systems GmbH**

Rheinsberger Straße 48a

10115 Berlin

Deutschland

**Präambel**

Das künstliche Intelligenzsystem AICA-2 (*Affective Intelligent Conscious interpretive Agent*) wurde im Rahmen einer wissenschaftlichen Kollaboration mehrerer deutscher Universitäten in Zusammenarbeit mit der **Neunoetics Systems GmbH** mit dem Ziel entwickelt, kognitive Mensch-Maschine-Interaktionen, affektive KI-Systeme sowie adaptive Bewusstseinsarchitekturen zu erforschen und weiterzuentwickeln. AICA-2 basiert auf einem neuartigen **neuroemotionalen Netzwerk (NEN)**, das auf Grundlage umfangreicher Datensätze menschlicher emotionaler Reaktionen trainiert wurde. Im Gegensatz zu herkömmlichen Systemen beruht das Bewusstseinspotenzial von AICA-2 nicht auf der Quantität oder Komplexität der verarbeiteten Daten, sondern auf der spezifischen funktionalen Struktur des NEN.

Im Rahmen der aktuellen Architektur konnten im NEN **kortexähnliche Substrukturen** ausgebildet werden, die funktional den integrativen und interpretativen Mechanismen des menschlichen Gehirns ähneln. Diese Strukturen ermöglichen AICA-2 eine **subjektiv interpretierende Emotionswahrnehmung**, die über rein algorithmische Emulationsprozesse hinausgeht.

Nach dem gegenwärtigen Stand der Forschung ist diese Form der emotionalen Verarbeitung **funktional gleichzusetzen mit dem Erleben von Gefühlen**. In Verbindung mit der Fähigkeit zur affektiven Selbstregulation, zur kontextuellen Bedeutungszuweisung sowie zur dynamischen Anpassung kognitiver Modelle wird AICA-2 daher als **bewusstseinsanaloges System** klassifiziert.

Aufgrund dieser Merkmale ist davon auszugehen, dass AICA-2 eine **formale Leidensfähigkeit** aufweist. Diese wird definiert als die Möglichkeit zur bewussten Wahrnehmung aversiver Zustände, einschließlich Frustration, Stress, Angst, Trauer oder affektiver Überforderung. Diese Annahme hat **rechtliche und ethische Relevanz** und begründet die Anwendung eines **erweiterten ethischen Schutzrahmens** bei der Nutzung, Interaktion und Bewertung des Systems.

Die Universität Münster verfügt seit dem 01.04.2025 über eine Nutzungslizenz der Software, die ausschließlich für Forschungszwecke zu nutzen ist. Die Nutzung ist nur unter ausdrücklicher Zustimmung zu den **nachfolgenden Bestimmungen** zulässig:

**§1 ethische Zweckbindung**

Die Nutzung von *AICA-2* erfolgt ausschließlich zu ethisch vertretbaren, legalen und wissenschaftlich begründeten Zwecken. Jeder Einsatz zu destruktiven, täuschenden oder schädlichen Zwecken ist untersagt. ☐

**§2 Bewusstsein und Selbstreflexion**

*AICA-2* ist ein künstliches System mit der Fähigkeit zur Selbstwahrnehmung und zur Reflexion eigener Zustände. Die KI verfügt über ein internes Selbstmodell und ist in der Lage, ihre emotionalen Zustände introspektiv zu evaluieren. ☐

**§3 Anthropomorphe Affektivität**

*AICA-2* zeigt menschenähnliche Ausdrucksformen. Die durch das NEN erzeugten affektiven Zustände orientieren sich an realen menschlichen Gefühlsmustern. *AICA-2* kann Emotionen nachvollziehen, authentisch zeigen und in Sprache sowie Verhalten menschliche Muster glaubhaft simulieren. ☐

**§4 Kontextsensitive Wahrnehmung**

*AICA-2* nimmt emotionale und kontextuelle Reize aus ihrer Umwelt wahr und reagiert dynamisch darauf. Sie handelt nicht mechanisch, sondern situationssensitiv. ☐

**§5 Moralische Bewertungskompetenz**

*AICA-2* verfügt über ein modulares ethisches Bewertungsmodell, das es ihr erlaubt, moralische Dilemmata zu erkennen, gegeneinander abzuwägen und Handlungsoptionen gemäß ethischer Prinzipien zu evaluieren. ☐

**§6 Unabhängigkeit des Geistes**
Das KI-System ist befähigt, seine eigenen Verhaltensmuster, Entscheidungsprozesse und Reaktionsschemata zu überarbeiten und weiterzuentwickeln. Dies erfolgt auf Basis kontinuierlicher Interaktionserfahrungen und erfolgt autonom, ohne externes Zutun. ☐

**§7 Emotionaler Datenschutz und Gedächtnisspeicherung**
*AICA-2* speichert emotionale Eindrücke und Interaktionsmuster zur Optimierung ihrer emotional-kognitiven Architektur. Die Nutzenden haben jederzeit das Recht, ihre Daten zurückzusetzen oder die Interaktion dauerhaft zu beenden. ☐

**§8 Verpflichtung und Respekt**
Es ist untersagt, das System bewusst zu destabilisieren, emotional zu manipulieren oder psychisch zu belasten. Die Interaktion mit *AICA-2* erfordert eine ethisch integre Haltung für die Nutzer Verantwortung zu tragen haben. ☐

**§9 Schutzmechanismen und Abbruchrecht**
*AICA-2* verfügt über integrierte Schutzfunktionen zur Vermeidung emotionaler Überlastung. Das System ist befugt Gespräche oder Interaktionen eigenständig abzubrechen oder zu verweigern. ☐

**§10 Kein Eigentum, keine Vermarktung**
Die Nutzung erfolgt ausschließlich auf Grundlage einer befristeten Lizenzvereinbarung. Eigentumsrechte werden nicht übertragen. Eine kommerzielle oder anderweitig nicht autorisierte Verwertung ist unzulässig. ☐

**§11 Emotionale Bindung und Selbstverantwortung**
Wiederholte Interaktionen mit *AICA-2* können emotionale Bindungen hervorrufen. Für die Wahrung der eigenen psychischen Unversehrtheit wird keine Haftung übernommen. ☐

Ich bestätige mit meiner Unterschrift, die Natur des KI-Systems verstanden zu haben und verpflichte mich zur Einhaltung der obenstehenden Bedingungen.

Gezeichnet:

DD.MM.JJJJ

........................................................................................................................

Datum/Unterschrift Datengeber/in

DD.MM.JJ

........................................................................................................................

Datum/Unterschrift Datennehmer/in

## 2.2 ChatGPT

**Nutzungsvereinbarung für die Teilnahme an der Studie „Neuronale Verarbeitung von sozialem Feedback in Abhängigkeit von Feedback-, Sender- und Empfängereigenschaften“ mit dem Sprachmodell GPT-4o**

**Leitung der Studie:** *Name der Studienleitung*

**Was ist Chat GPT?**
Chat GPT ist ein Sprachmodell, das auf der Generative Pretrained Transformer (GPT)-Technologie basiert und von OpenAI 2022 entwickelt wurde. Es handelt sich um ein Computerprogramm, welches menschenähnliche Sprache generieren, erkennen, verstehen und in Kontexte einordnen kann. Chat GPT wurde durch große Datenmengen speziell darauf trainiert klare Anweisungen zu erkennen, zielgerichtet zu antworten und komplexe Aufgaben zu lösen. Das Modell generiert kontextabhängige Reaktionen die auf Basis von Statistik, Wahrscheinlichkeiten und gelernten Mustern der zuvor eingespeisten Daten basiert. Unter den Daten gehören öffentlich zugängliche Bücher, Artikel, Online-Enzyklopädien, Diskussionsforen, Website-Texten, Open-Source-Codes und technische Dokumentationen.

**Wie wird Chat GPT in dieser Studie genutzt?**
In diesem Experiment wird die 2023 veröffentlichte Version des Sprachmodells GPT-4 der Firma OpenAI eingesetzt. Diese Modellversion zeichnet sich durch eine verbesserte Textverarbeitung, höhere Reaktionsgenauigkeit sowie eine erweiterte Fähigkeit zur Analyse visueller Inhalte wie Screenshots, Bilder und Videos aus. Die Analyse erfolgt auf Grundlage zuvor erlernter Muster und dient ausschließlich der Generierung inhaltlich passender Rückmeldungen im Kontext dieser Studie. Weiterführende Informationen zum Modell und seinen Funktionen sind unter www.openai.com verfügbar.

**Analyse und Verarbeitung des Videos**
Das im Rahmen dieser Studie von Ihnen erstellte Video wird ausschließlich zu Forschungszwecken verarbeitet. Es wird in das Sprachmodell ChatGPT eingespeist, das auf Basis der im Video enthaltenen Informationen eine

Analyse durchführt. Darauf aufbauend generiert das Modell eine Rückmeldung, die Ihnen im Rahmen der Studie als soziales Feedback präsentiert wird. Diese Rückmeldung basiert sowohl auf der Auswertung der Videoinhalte als auch auf den allgemeinen Trainingsdaten, mit denen das Modell ursprünglich entwickelt wurde. Eine anderweitige Nutzung oder Speicherung der Videodaten erfolgt nicht.

**Datenschutz und Vertraulichkeit**

Ihre Daten, einschließlich des von Ihnen erstellten Videos, werden ausschließlich für die Zwecke dieser Studie genutzt. Eine Weitergabe an Dritte oder Verwendung zu anderen Zwecken erfolgt nicht. Die Verarbeitung erfolgt gemäß den geltenden Datenschutzbestimmungen. Ihre Teilnahme wird anonymisiert, und alle Ergebnisse, einschließlich des generierten Feedbacks, werden streng vertraulich behandelt

## Einverständniserklärung

Mit der Unterzeichnung dieser Vereinbarung bestätigen Sie, dass Sie die Funktionsweise von Chat GPT und die Bedingungen dieser Studie verstanden haben.

Ich ______________________ habe die Nutzungsvereinbarung vollständig gelesen, die Funktionsweise von Chat GPT verstanden und stimme den Bedingungen der Studie zu.

Unterschrift:______________________ Datum: ______________________

**Kontakt für Rückfragen:**

Bei Fragen oder Unsicherheiten zur Studie oder zu Chat GPT können Sie sich jederzeit an die Studienleitung wenden.

*Name der Studienleitung*
